# ULK1 and ULK2 Restrain Skeletal Myofiber Growth by Balancing Protein Synthesis and Degradation

**DOI:** 10.64898/2026.09.06.749722

**Published:** 2026-09-10

**Authors:** Wangkuk Son, Jordan Fuqua, Matthew P. Harris, Ryan J. Allen, Ana Kronemberger, Julia Hughes, Luis G. O. de Sousa, Leonid Zingman, Sue C. Bodine, Benjamin F. Miller, Vitor A. Lira

## Abstract

**Background:**

Skeletal muscle is vital for mobility and metabolic regulation, impacting independence and overall health. Increases in skeletal muscle mass and contractile function during development, and their maintenance during adulthood and aging, rely on an intricate coordination between protein synthesis and degradation processes that remains incompletely understood. Here, we investigated a potential role for the autophagy-initiating kinases ULK1 and ULK2 in broadly modulating protein metabolism in skeletal muscle.

**Methods:**

Studies were conducted in young (4-6 wk-old) and adult (7-10 mo.-old) mice with skeletal muscle-specific knockout of *Ulk1* and *Ulk2* (i.e., Ulk1/2 ^skmDKO^ ) and wild-type littermates (WT). Short-term deficiency of these proteins was achieved via electroporation of plasmids (encoding specific microRNAs targeting *Ulk1* and *Ulk2* ) into muscles of 4 mo.-old wild-type mice. Protein metabolism was assessed via deuterium oxide (D _2_ O) labeling, whereas anabolic signaling was investigated under insulin and leucine administration.

**Results:**

Lifelong *Ulk1/2* deficiency markedly impaired autophagy flux (i.e., LC3-II accumulated with colchicine treatment only in wild-type mice, P<0.001), compromised muscle quality, as evidenced by an increase in centrally nucleated fibers (from 0.1% to 4.5% in females, and from 0.8% to 22.7% in males (P<0.001), primarily involving MyHC type 2b fibers) and impaired force of dorsiflexors and plantar flexors in males (20%, P<0.01), and plantar flexors in females (24%, P<0.01). Despite these deficits, *Ulk1/2* deficiency promoted robust muscle hypertrophy, evidenced by increased diameters of all major MyHC fiber types in the tibialis anterior and soleus muscles (i.e., by 10-15% in males, and 14-20% in females, P<0.05). Short-term deficiency (up to 4 weeks) of *Ulk1/2* in adult skeletal muscle, however, led to myofiber hypertrophy (13%, P<0.05) without impairments in force or changes in central nucleation of fibers, pointing to an initial period of muscle quality preservation. Mechanistically, *Ulk1/2* deficiency led to elevated myofibrillar protein synthesis (23% higher Ksyn, P<0.05) and decreased mitochondrial and sarcoplasmic protein degradation (16% and 14% lower Kdeg, P=0.09 and P<0.05, respectively). Further mechanistic studies revealed that hypertrophy was accompanied by enhanced mTORC1 activity independent of AKT in *Ulk1/2* -deficient muscle.

**Conclusions:**

These results indicate that ULK1 and ULK2 jointly sustain autophagy and limit mTORC1-driven protein synthesis to govern skeletal muscle protein metabolism, with lifelong deficiency increasing muscle size at the expense of quality and function, while short-term deficiency permits hypertrophy without impairment. These findings identify ULK1/2 as a novel node coordinating protein turnover in skeletal muscle, warranting investigation as a therapeutic strategy for atrophy and weakness.

## Full Text Availability

The license terms selected by the author(s) for this preprint version do not permit archiving in PMC. The full text is available from the preprint server.

